# Comparison of US and EU Prices for Orphan Drugs in the Perspective of the Considered US Orphan Drugs Act Modifications and Discussed Price-Regulation Mechanisms Adjustments in US and European Union

**DOI:** 10.3390/ijerph191912098

**Published:** 2022-09-24

**Authors:** Paweł Żelewski, Michał Wojna, Katarzyna Sygit, Elżbieta Cipora, Izabela Gąska, Mateusz Niemiec, Mateusz Kaczmarski, Tomasz Banaś, Beata Karakiewicz, Artur Kotwas, Paulina Zabielska, Olga Partyka, Monika Pajewska, Edyta Krzych-Fałta, Ewa Bandurska, Weronika Ciećko, Aleksandra Czerw

**Affiliations:** 1 Department of Economics, Kozminsky University, 03-301 Warsaw, Poland; 2 Department of Health Economics and Medical Law, Medical University of Warsaw, Żwirk i Wigury 81 St., 02-091 Warsaw, Poland; 3 Faculty of Health Sciences, Calisia University, 62-800 Kalisz, Poland; 4 Medical Institute, Jan Grodek State University in Sanok, 38-500 Sanok, Poland; 5 Department of Gynaecology and Oncology, Jagiellonian University Medical College, 31-501 Cracow, Poland; 6 Department of Radiotherapy, Maria Sklodowska-Curie Institute-Oncology Centre, 31-115 Cracow, Poland; 7 Subdepartment of Social Medicine and Public Health, Department of Social Medicine, Pomeranian Medical University in Szczecin, 70-204 Szczecin, Poland; 8 Department of Economic and System Analyses, National Institute of Public Health—NIH—National Research Institute, 00-791 Warsaw, Poland; 9 Department of Basic of Nursing, Faculty of Health Sciences, Medical University of Warsaw, 01-445 Warsaw, Poland; 10 Center for Competence Development, Integrated Care and e-Health, Medical University of Gdańsk, 80-204 Gdansk, Poland

**Keywords:** orphan drugs, price, pharmacology, economy

## Abstract

The 2019 worldwide sales of Orphan Drugs were estimated at $136 billion USD, which constituted 16% of the global pharmaceutical prescription market and is expected to grow by 12% in the next 5 years. A better understanding of Orphan Drug pricing may contribute to on-going discussions on Orphan Drug Act (ODA) corrections in US or modifications of price setting mechanisms in EU. The objective of the study was comparison and analysis of the prices of Orphan Drugs in US and EU. All drugs with Orphan Drug status were compared in the US and EU. For the US prices, the US Department of Veterans Affairs (VA) was sourced. The EU List Prices came from six EU countries: Denmark, France, Germany, Greece, Poland, Spain. We found US prices to be higher than the six selected EU countries. The average Price Ratio was 1.64. The prices across EU countries were more homogeneous, while the number of the reimbursed and therefore available to patient medicines varied and was correlated with GDP per capita r = 0.87. Considered implementation of the External Reference Price system in US may generate significant savings in the US but may result in upward pressure on pricing of Orphan Drugs in EU. Centralization of the Orphan Drugs pricing negotiations in EU may prevent such development and offer a win-win opportunity for all involved parties.

## 1. Introduction

The rapidly emerging class of Orphan Drugs brings hope and new treatment opportunities for patients suffering from rare diseases for which there is often no other treatment available. Unfortunately, patients suffering from rare diseases have limited treatment options which only intensifies health inequalities and drugs availability differences among countries [1,2,3]. 

On the other hand, introduction of new Orphan Drugs to the market is the key driver of the overall spending growth on drugs and set significant financial challenges for health care systems. The 2019 worldwide sales of Orphan Drugs were estimated at $136 billion USD, which constituted 16% of the global pharmaceutical prescription market [4]. Observed spectacular sales growth of Orphan Drugs is expected to continue globally at the rate of over 12% per year (CAGR 2019–2024), with sales forecasted to reach $242 billion USD and 20.3% of the worldwide prescription market in 2024 [4]. In the period of 2010–2016, the growth of Orphan Drugs in Europe was evaluated at 16% per year (CAGR), much higher than overall market growth or earlier forecasts [5]. Similarly, the share of Orphan Drugs among all newly approved drugs by the FDA grew from 23% from 1998 to 2007 to 42% in 2008 to 2017, with 34 new Orphan Drugs registered in 2018, which constituted 58% of all new approvals [6], while between 2011 and 2016 the number of Orphan Drugs approved for use in EU grew by 18% per year [7]. 

The term Orphan Drugs is reserved for drugs designed to treat rare diseases. Rare diseases are defined in the EU as diseases affecting less than 0.05% of the population or in the US less than 200,000 people. The spectacular growth of the number of the Orphan Drugs, is generally recognized as the big success of the US Orphan Disease Act (ODA) passed in 1983 and the similar EU Orphan Regulation (No 141/200) introduced in 2000. Those regulations shifted attention and resources of the drug companies toward rare diseases. However, there are concerns about some of the undesired and unexpected effects of the ODA, and discussions about proposed corrections are growing and becoming more intense. Considered options focus on changes in definition of Orphan Drugs directed primarily to limit “salami slicing” practices, limitation of the financial incentives for orphan drugs development, or different models of price-setting mechanisms [8,9,10]. The discussion around Orphan Drugs is also very active in EU, but details differ from those in the US. The EU discussion is rather focused on the “excessive pricing” of those drugs, resulting in limited public access to novelty therapies rather than on modifications of the regulatory process [11,12]. 

A September 2019 report prepared by the Ways and Means Committee, a US House of Representative committee, found US prices nearly four times higher than average prices in comparative countries and indicated External Reference Pricing (ERP) as the mechanism which could generate up to USD 49 billion savings in Medicare Part D alone [13]. Considered for implementation ERP linking US pricing to prices in countries with similar economic conditions (using GDP per capita as primary indicator) and referring directly to the EU situation may significantly impact pricing strategies executed by international pharmaceutical companies in EU. Discussed in the US, changes are triggered by fast growth of the US expenditure on specialty drugs, including Orphan Drugs. In the US, the total national expenditure on drugs, reached USD 457 billion in 2016, with a 27% growth since 2011. This growth was predominantly driven by a rise in spending for specialty drugs, covered by Medicare Part D and Medicaid programs [14,15]. According to the Centers for Medicare and Medicaid Services (CMS) and IQVIA, total US national prescription drug spending growth was 25.7% from 2013 to 2017, which was attributed to the introduction of expensive specialty drugs, and it is expected to grow from USD 333.4 billion in 2017 to USD 435 billion in 2023 [16,17]. Although the specialty drug term is not unambiguously defined, according to the commonly used IQVIA definition, 87% of Orphan Drug spending falls into this category [18]. 

Orphan Drug status may change over time and is granted to the specific indication of the drug, not to the drug itself. Therefore, out of the total number of 503 Orphan Drugs approved in the US as of August 2018, a total of 394 (78%) had an orphan-only indication and 109 (22%) had both an orphan and non-orphan indication. This distinction is important, as the spending on orphan-only indications constitutes just over one third of the total spending on all in this group. Spending in 2017 on orphan-only indications was USD 43 billion, which constituted 9.6% of total drug spending with growth of 11.4% CAGR over 5 years. While the same drugs in orphan and non-orphan indications generated spending of USD 112 billion, which constituted 24.9% of the overall drug spending and contributed very significantly to the spending growth of the specialty treatments and the overall market [18]. As a result, although a majority of sales were generated in non-orphan indications, 7 out of 10 drugs with the biggest global sales in 2017 were approved by the FDA with orphan indications [19,20]. 

In the EU, according to the recently published study, the combined sales of Orphan Drugs in eight EU countries (Austria, Belgium, Germany, Spain, France, Ireland, Italy, UK), constituting jointly 77% of the EU GDP and 68% of the population, reached in 2017 the level of EUR 10.5 billion with 7.2% market share and growth of 16% per year (CAGR) in the period of 2010–2017 [5]. The growth of the total pharmaceutical market in this period reached only 3%, which was predominantly explained by the increasing volumes of the lower priced generics and biosimilars compensating growing expenses on Orphan Drugs [5]. As the growth of expenditures on Orphan Drugs is expected to continue, the healthcare policy makers need to carefully consider Orphan Drugs funding and management, to ensure wide public access to these unique products. The spectrum of the public proposals discussed in EU targeted to decrease prices and increase patients access to Orphan Drugs include modifications of the used pharmacoeconomic evaluation techniques [21,22], coordination of the reimbursement and pricing negotiations on the EU level [23], and increased support and extensive use of the non-profit organizations in search for new Orphan Drugs [24]. 

Aggressive pricing of Orphan Drugs with annual therapy costs commonly above 100,000 USD per one patient [20], explained by manufacturers as resulting from the growing costs of research and development [5,23], are key contributor of the growing expenses on drugs. 

### 1.1. Study Objectives

Considering the above, we took the initiative to compare prices of Orphan Drugs from two biggest global pharmaceutical markets: the US and EU, providing jointly more than 2/3 of the global total R&D investment into innovative medicines and forming a dominant part of the total global market for the innovative treatments. While the research available until today comparing US drug prices to EU prices were performed on the variously defined samples of medicines from various segments (generics, branded products, or cancer treatments) [24,25,26,27], without special focus put on Orphan Drugs, the goal of our study is to be the first to compare prices of this specifically defined segment. Prices of Orphan Drugs are agreed through the negotiations process between the manufacturer and the payers. While the manufacturers operate and negotiate from a global perspective, highly fragmented payers—in EU separate public health authorities for each country, in US various private insurers and public institutions—negotiate from a much narrower local perspective. This situation has serious impact on applied pricing strategies; in EU, payers use more and more various cross-country price reference tools, currently under discussion also in the US, while the manufacturers tend to develop highly structured strategies, prioritizing the markets and order of negotiations, frequently choosing to limit the number of countries or markets where the new treatment is made available for patients rather than to provide additional price concessions requested in economically weaker countries. Therefore, in the attempt to provide some insight into the extent and effects of such strategies, we also performed some additional price comparisons in different sub-groups of Orphan Drugs. First, we divided all analyzed Orphan Drugs into two groups: “Single and Identical Pack Only” defined as drugs marketed in all analyzed countries in only one and identical pack (single pack type with same dose, number of units, concentration, etc. but could have different brand name) and “Other as Single and Identical Pack Only” for all remaining analyzed drugs. We were interested to test if the eventually found cross-country price differences in the easier to compare “Single and Identical Pack Only” group were smaller than in the more difficult to compare “Other as Single and Identical Pack Only”.

Secondly, we divided all analyzed drugs based on the year in which they were approved by the FDA. To achieve comparative group sizes, the first group contained drugs registered by the FDA in years 1996–2014 and the second in years 2015–2019. As in both groups we compared the most recent 2019 prices, the goal of our analysis was to test if the longer time on the market (longer time from initial price negotiations) could result in higher price differential. If so, it could suggest that the cross-country price strategy coordination by the manufacturers was getting stricter over time or that for drugs with longer presence on the market, the price flexibility presented by manufacturers was increasing and was more responsive to customer demands. 

We have also attempted to verify if the number of Orphan Drugs with agreed price and therefore available for patients in the particular country could be related to some general macroeconomic indicators. In particular, we decided to test the correlation between the number of Orphan Drugs with agreed price, which was in our analysis synonymous with granted reimbursement and availability to patients, and the value of the nominal GDP per capita. In the perspective of our constrained resources, we decided at this stage to limit our analysis to this macroeconomic variable, leaving many other potential variables—for example, epidemiology, demographic, population size, healthcare expenditure, share of expenses on medicines, specific market control mechanisms—as the potential field for future research.

As Orphan Drugs constitute not only the biggest and the fastest-growing segment in terms of the industry R&D investment, but also is the source of the fastest growth of the general spendings on pharmaceuticals and generate highly emotional public discussion on the costs of the individual treatments commonly exceeding hundred thousand USD per individual patient per year of treatment, we considered our analysis as the valuable support for the on-going discussions and negotiations between payers, health authorities, and the industry. Those negotiations not only determine the immediate patients’ access to the specific innovative treatment, but also determine longer-term R&D investment decisions of the industry into rare diseases, which are currently recognized as the main field of unmet medical needs and remain in public focus. We hope that the provided insight may help not only in on-going discussions about potential ODA modifications or price-control mechanisms in the US and EU but may also strengthen the base for the development of the theoretical models and recommendations for the drug policy, facing the reality where the new drugs development investments are centralized and performed in the global perspective, while the return on this investment is determined by highly fragmented local pricing/access decisions. Thorough understanding of the international pricing reality in the fastest growing segment of the newly developed medicines combined with the understanding of the patients’ access limitations to those newly emerging treatments may help in defining win-win strategies, allowing to stimulate R&D processes, control the financial burden on payers while expanding, and accelerating public access to the novelty treatments. Obviously, developing and implementing such strategies sounds like a very ambitious goal requiring thorough understanding of the situation and much wider cooperation of all involved parties; still, we believe that such goals are achievable, and we hope that our study may help to stimulate the already on-going discussions in this field. We would be highly satisfied to see our publication inspiring for further systematic investigations in this area.

### 1.2. Methodology—General Considerations and Selected Approach

Price comparison of pharmaceuticals across different markets is not easy, primarily due to limited data availability, non-disclosed data on rebates, confidential agreements with payers, and the multiple sizes and strengths of the marketed drugs presentations.

Although in EU, the officially agreed upon List Prices for reimbursed drugs are generally publicly available, the information on the negotiations in each country between manufacturers and price-setting authorities and their rebates (sometimes granted to individual hospitals) or other cost-containment measures are typically not publicly disclosed. From the countries selected for our comparison of 6 EU countries, only Germany officially publishes the level of the obligatory rebates, which equals 7% calculated from List Prices. We were also able to estimate the value of the average additional rebate given on all Orphan Drugs in 2018 in Poland at 17%. 

The situation is even more complicated in the US. Due to decentralized health care and health insurance model, the prices offered to different customers for the same drug may be significantly different, with various structures of potential rebates applied on different levels of the distribution or insurance structures. As the data on paid prices is generally not publicly available, researchers commonly use publicly available Average Wholesaler Prices (AWP) or Wholesaler Acquisition Costs (AWC) for comparison. These originate from drug manufacturers and refer to selling prices before any rebates. In the next steps, these are adjusted for the approximated level of the market rebates, evaluated based on findings from different Medicare and Medicaid reports, and specially designed public and independent research. 

In our comparison, we decided to use the List Prices for EU. For the US, we followed the approach recently proposed by researchers from the University of Maryland and the University of Wisconsin–Madison and to use prices set with the US Department of Veteran Affairs (VA) as the lower bound of the applicable to US drug prices [28]. Our decision was driven by two main reasons. The first reason is the public availability and unambiguity of available pricing data. Second, as the EU List prices are the highest possible prices in EU (excluding potential additional rebates) and as the VAFSS prices are considered as the lowest prices in the US, our comparison allowed to find the minimum level of price difference in case the prices of the US were higher than EU prices, which we assumed as the initial hypothesis based on the review of the other publications. VA agrees to negotiations in two price levels: a basic price level granted to all federal direct health care payer agencies (FSS) and additionally a discounted price offered to the biggest federal purchasers (Big 4). By definition, the FSS price must not be higher than the lowest net price (after all rebates) charged by the manufacturer to their most favored nonfederal customer under similar market conditions. While the Big 4 price must be at minimum 24% discounted from the nonfederal average manufacturer price, defined as the weighted average price of a single form and dosage unit, paid by wholesalers to a manufacturer, taking into account cash discounts or similar price reductions [29]. The lower of those two prices (VAFSS) are commonly considered to be the lowest prices on the US market [30]. 

In another study comparing published price data by drug manufacturers in 2016, transparency reports with VAFSS prices concluded that the average discounts may be substantially higher than commonly evaluated. Therefore, VAFSS prices may be a relatively close approximation of the average discounted prices paid in reality to manufacturers by Pharmacy Benefits Managers (PBMs) or third-party payers in the US [31]. 

The EU registration of Orphan Drugs is fully centralized by the European Medicines Agency (EMA), while pricing and reimbursement are the exclusive responsibilities of the individual countries. The reimbursement and pricing systems in EU are defined and operated in each EU country separately, but with significant similarities across the majority of them. Typically, prices are being set in the reimbursement negotiations process conducted between manufacturer and public health care institutions. In the majority of the EU countries, pricing for non-reimbursed drugs is free. Still the significant use of the high-cost, specialty medicines including Orphan Drugs is in practice conditioned by reimbursement. Agreed upon reimbursement negotiations prices are legally binding for all trades within the territory of the country. Therefore, the presence of the given drug on the reimbursement list and the availability of the set price were used in our study as the determinants for the classification of the given Orphan Drug as available for patients use in the country. Although such methodology gives only a limited base for cross-country comparison as the access to the treatment may be further limited through other measures such as prescription limitations to certain groups of physicians or treatment centers, centralization of the individual treatment decisions on the national level or financial budget limitation set for specific treatments, we considered this methodology as a good starting point for further, more precise evaluations.

## 2. Materials and Methods

For the US/EU comparison we analyzed and compared Orphan Drug prices from the selected 6 EU countries with the US prices. Independently, we have also compared prices from each EU country versus the average from those countries calculated for each product individually.

The selection of the EU countries was determined by our willingness to include EU countries with different levels of economic power, population size, geographical location, pharma industry profiles, health care sophistication, and EU political or regulatory experience. In our opinion, included in the analysis Germany, France, Denmark, Spain, Greece, and Poland jointly forming 50% of the EU population and GDP, represent a well-differentiated spectrum of countries and a good representation of the entire EU. 

For the drug selection, we compared the list of the 139 approved by the European Medicines Agency (EMA) with Orphan Drug status, which was sourced from the EMA internet site available to the general public online [32]. The list of the 593 drugs approved by FDA for the use in US with the Orphan Drug status (status 31 December 2019) was sourced from the FDA’s publicly available site [33]. Both lists were compared, and 104 drugs with Orphan Drug status granted by both, EMA and FDA, were identified. Further we have decided to exclude from further analysis 7 drugs (*Dacogen*, *Exjade*, *Orfadin*, *Ravicti*, *Siklos*, *Votubia/Afinitor*, *Xaluprine/Purixan*) with generics available in at least one of the compared countries (to eliminate price differences caused by the different market exclusivity status), four drugs for which the price with the Department of Veteran Affairs was not set on the date of the data sourcing (*Coagadex*, *Gliolan*, *Litak/Leustatin*, *Scenesse*) and two drugs for which the price was not set on the date of the data sourcing in any of the analyzed EU countries (*Mepsevil*, *Poteligo*). Additionally, to avoid bias potentially caused by the presence of the different galenic form (oral, iv) in different countries, we separated for the analysis oral and iv forms of 2 products (Cresemba, Prevymis). All analyzed Orphan Drugs were segmented in two ways. Firstly, we have identified two groups: “Single and Identical Pack Only”, defined as drugs marketed in all analyzed countries in only one and identical pack (same dose, number of units, concentration, galenic form, but sometimes with different brand name), and “Other as Single and Identical Pack Only” for all remaining ones. Secondly, we divided all analyzed drugs based on the year in which they were approved by the FDA. To achieve comparative group sizes, the first group contained drugs registered by the FDA in years 1996–2014 and the second in years 2015–2019. The final list of 93 Orphan Drugs analyzed by us and used by us segmentation is presented in Table 1.

For price comparisons within the EU countries, we have used published official List Prices, while for the US we have used VAFSS prices defined as the lower from the two price levels (FSS and Big 4) set with US Department of Veteran Affairs (VA). Compared price data was collected from the publicly available reputable sources Table 2.

Our analysis was performed in two parallel processes, first comparing US and EU prices, second comparing prices from the individual EU countries vs. the average from those six EU countries.

All available pricing data were consolidated in a single Excel file, separately for each country and each pack type. Any difference in presentation (tablet/capsule/solution), active substance dose per tablet/capsule, ml of solution, volume of the solution, or number of tablets/capsules/vials in the pack was treated as determining different “pack type” of the drug. Prices from Poland (PLN) were recalculated to EUR and EUR prices were recalculated to USD using the average 2019 exchange rate of EUR/PLN = 4.26 and EUR/USD = 1.12. All EU prices were analyzed at the ex-manufacturer price level, free of any potential taxes or distribution margins. As in Poland published list prices included VAT (8%), we recalculated those prices to net prices.

In the first step of our analysis, we consolidated and converted to US Dollars all of the available pricing data, separated for each pack type in one Excel file. Different pack types were defined based on any difference in presentation (tablet/capsule/solution), dose per tablet/capsule, concentration per ml, volume of the solution, or number of tablets/capsules/vials in one pack. 

As the majority of the analyzed drugs were marketed in the EU in different strengths/pack sizes than in the US, a simple comparison of prices was not technically possible. Therefore, for each country and pack type in the database, we separately calculated the quantity of the active substance in milligrams (mg) or international units (i.u.) as appropriate, followed with calculation of the price per 1 mg or 1 i.u. of the active substance. The lowest price per mg. or i.u. from all pack types for each Orphan Drug in each country was selected and used for further Price Ratio calculations. For the US/EU comparisons, Price Ratio was defined as the ratio between the lowest US VAFFS price per mg/i.u and the lowest EU Country List price per mg/i.u., while for the EU internal comparison Price Ratio was defined as the ratio between the lowest price per mg/i.u in the analyzed country and the average from all available lowest prices per mg or i.u. from the six analyzed EU Countries. For three drugs (Lutathera, Kymriah, and Yescarta) where the mg or i.u. measurements were not applicable, but the drugs were marketed in the single and the same pack type in all countries, we have calculated Price Ratios directly from the pack prices. 

In the following step, the average combined US/EU Price Ratios were calculated separately for each of the 6 EU individual countries in the defined earlier segments as the average from the appropriate for the segment price ratios calculated for this country. The average Price Ratios were also calculated in all segments defined by us for US/6 EU countries jointly, as the average from the appropriate for the analyzed segment price ratios calculated for all 6 EU countries jointly.

Price Ratios for US/EU individual countries, US/6 EU countries jointly, and EU internal comparisons were analyzed and compared among each other in defined segments and tested vs. assumed hypothesis of “no price difference” with the use of the *t*-test, one-sample, assuming unequal variances.

We have also compared the number of reimbursed Orphan Drugs (with set List Prices in each of the analyzed EU country) among all analyzed countries and tested this for the eventual relationship with the nominal GDP value per capita (Statista, 2019).

## 3. Results

### 3.1. US vs. EU Comparison

The number of compared drugs, pack types and values of calculated Price Ratios are presented in Table 3 below. 

VAFSS US prices were on average 1.64 times higher than the List Prices of the same drugs in compared countries (range from 1.46 for Denmark to 2.11 for Poland). All Average Price Ratios calculated for the six EU countries separately or jointly were significantly higher compared to “no US/EU price difference” hypothesis (*p* < 0.005, *t*-test, single-sample vs. assumed = 1, unequal variances). The average from “Single and Identical Pack Only” Price Ratios of 1.43 (*n* = 90 all 6 EU countries jointly) was significantly lower compared to a ratio of 1.71 (*n* = 270) calculated for “Other as Single and Identical Pack Only” (*p* < 0.05, *t*-test two sample, assuming unequal variances).

**Figure 1:**
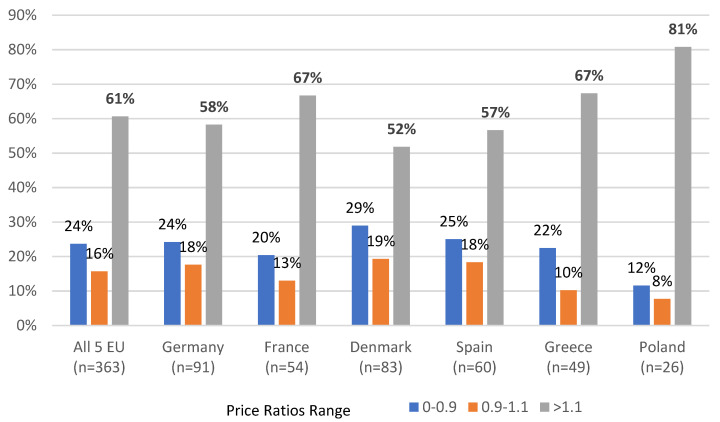
Distribution of VAFFS/EU Price Ratios per Country.

In 61% of cases, US VAFSS prices were higher than in other compared countries (from 52% in Denmark to 81% in Poland), for around 16% of brands prices were similar (defined as less than +/−10% price difference), and in 24% of cases, Orphan Drug prices were lower in the US (from 12% in Poland to 29% in Denmark) (Figure 1). 

**Figure 2:**
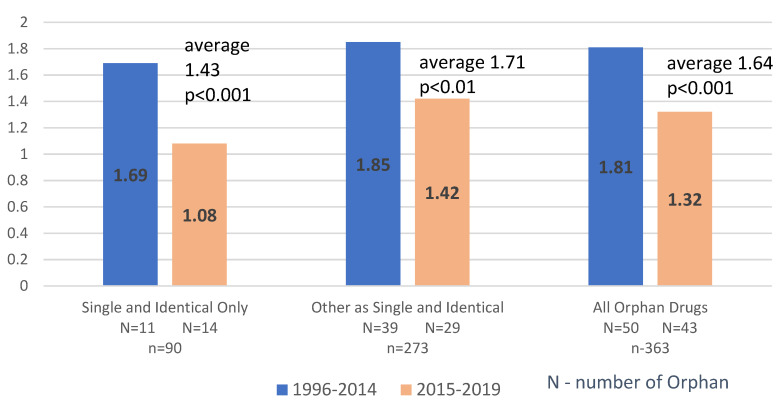
Orphan Drugs Average Price Ratios—segmented by the date of the first FDA approval. Comparison of US vs. six EU Countries (Denmark, France, Germany, Greece, Spain, Poland).

Average Price Ratios calculated for Orphan Drugs registered in the years 1996–2014 were significantly higher compared to Price Ratios from the years 2015–2019 in all subgroups; All Drugs: 1.81 vs. 1.32, Single and Identical Only 1.69 vs. 1.08, Other as Single and Identical Only 1.85 vs. 1.42 (*p* < 0.01). All except one (Single and Identical Pack Only, 2015–19, Price Ratio = 1,08; *n* = 39) Price Ratios were also significantly higher compared to “no US/EU price difference” hypothesis (*p* < 0.005) (Figure 2).

### 3.2. EU Individual Country vs. EU Average

**Figure 3:**
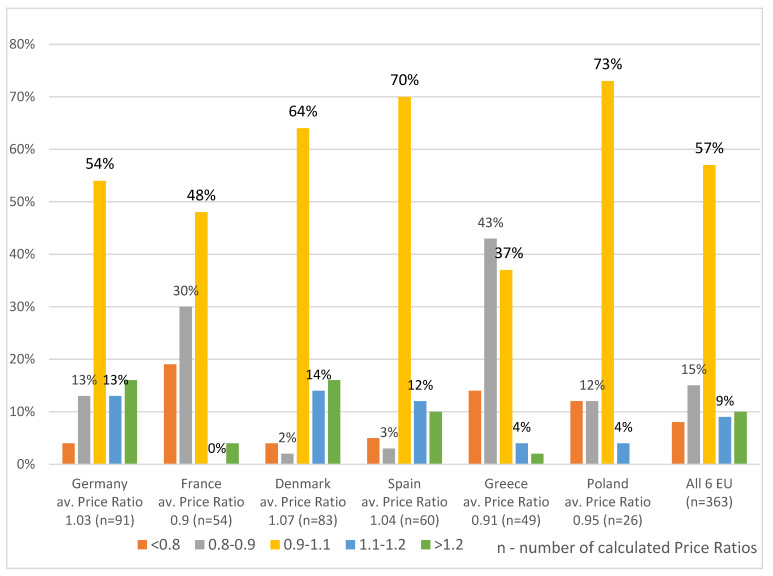
Price Ratios Distribution: Individual EU Country vs. average from six EU Countries (Denmark, France, Germany, Greece, Spain, Poland).

EU cross-country price comparisons of Orphan Drugs indicated much more homogenous pricing across all analyzed EU countries. Average Price Ratios calculated for each of the analyzed EU countries were in the range from 0.9 for France to 1.07 for Denmark. Similar results with Average Price Ratios in the range of 0.88 to 1.11 were observed in direct country-to-country cross-comparisons, which were limited to products available in both of the compared countries. Presented in Figure 3, distribution of the Price Ratios confirms the homogeneity of the Orphan Drug pricing across EU, with 57% of all price ratios being in the range of +/−10% from the six EU average and 82% in the range of +/−20%.

We have also observed that prices in the analyzed EU countries were more homogenous in the sub-group of the Orphan Drugs approved by FDA more recently or in the sub-group of “Single and Identical Pack Only” (Table 4). For example, 81% of the Price Ratios calculated in the sub-group of the “Single and Identical Pack Only” Orphan Drugs registered in the years 2015–2019 were in the range of 0.9–1.1 (95% in the range 0.8–1.2), while only 46% were in this range from the sub-group of the “Other as Single and Identical Pack Only” approved in years 2001–2014.

**Figure 4:**
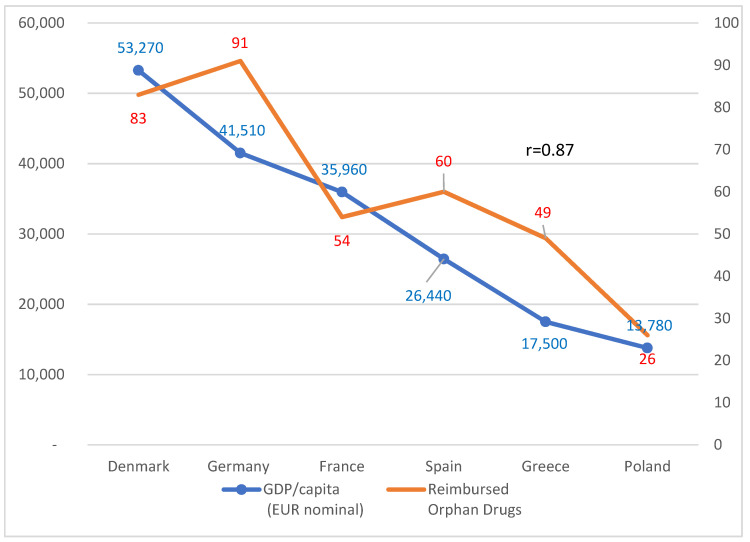
Number of reimbursed Orphan Drugs vs. GDP per capita in six EU Countries (2019). All Orphan Drugs with agreed List Price in the Country and reimbursed by public health insurance system are recognized as reimbursed. including drugs dispensed through open pharmacies. hospital pharmacies. or through special Drug Programs. GDP per capita values sourced from Eurostat.

The data presented in Figure 4 suggest a direct relationship between the number of reimbursed Orphan Drugs in the EU country and the GDP/capita. For countries presented in this paper. those two variables were strongly and positively correlated. (r = 0.87). A bigger sample size in the future would be beneficial to examine this relationship in detail.

## 4. Discussion

Although our study has several limitations to which we refer in more details in the following parts of the discussion. our analysis provides a strong base to make several observations. First. while the prices of Orphan Drugs appeared to be significantly higher in US than in EU. pricing of those drugs across EU countries was found to be highly homogeneous. Second. the price differentials are smaller for the drugs. which. due to its characteristics (single and identical form marketed globally). are easier to compare across the countries or/and were introduced into the market more recently. This observation seems valid for both comparisons performed in our study—US vs. EU and EU cross-country. Additionally. observed by us EU cross-country correlation between the number of the reimbursed and therefore available for patients in the given country Orphan Drugs and the value of this country GDP per capita. combined with the observation of the strong price homogeneity across EU. suggest that in the presence of the common for the EU reference pricing (cross-EU) the price flexibility demonstrated by the companies is limited and the drug manufacturers may rather opt to limit the number of EU countries where their innovative drugs are present than to increase price flexibility. Such a situation seems to be a logical consequence of the operated currently in the EU model. where international companies develop and follow single pricing strategy for the whole EU. while price/access negotiating counterparts are led by objectives set generally for the single country. Observations made by us also suggest that while the pricing strategy for Orphan Drugs seems to be very well coordinated by the industry across the EU countries. much less of such coordination is visible between EU and US until today. 

Our results indicating significantly higher prices of Orphan Drugs in US compared to EU are in line with other published price-comparison studies. Depending on the selection of the compared countries. analyzed segments of pharmaceuticals. or the price levels chosen for comparison. US prices were found to be from 1.8 to 4 times higher than international prices [13,30,34,35,36,37].

Outcomes of our research were also in line with the recently published findings indicating that US drug price differential vs. other countries tends to grow with the length of the market presence [30]. This may suggest that in the longer-term perspective. Orphan Drugs prices are managed more aggressively by the reimbursement authorities in EU compared to VA administration. Similarly. our observations indicate that Price Ratios for Orphan Drugs marketed in the single and the same pack in US and EU (which simplifies eventual cross-country price comparisons) results in significantly smaller or even no price difference for the drugs with a short market history (FDA approved 2015–2019). This is compared to much bigger differences for drugs approved earlier (1996–2014) thus leading to similar conclusions. 

Extrapolating on the EU findings. it may be expected that the currently considered introduction of the ERP mechanisms in the US is directly benchmarking EU prices. as recently presented in the 2019 Ways and Means Committee Report (US House of Representatives). combined with the currently observed significantly higher level of prices in US compared to EU. may help to control drug expenses in the US. but may as well create significant upward pressure on the future EU Orphan Drugs prices in EU. which could further limit patients’ access to the innovative treatments that are highly desired by them. In the perspective of the above. the search for the win-win solution for all stakeholders seems to become even more urgent and important. Already suggested public initiatives of the centralization of the pricing negotiations for innovative expensive treatments on the level of the entire EU may not only prevent such potential development but may also offer a win-win opportunity for all involved parties. EU-centralized Orphan Drugs price negotiations combined with the eventual acceptance by the payers and manufacturers of the significant effective price differences for the same drug across different “EU economic clusters” (combined with controlled distribution) could strengthen EU negotiation power. expand patient access to innovative treatments in poorer countries. and generate additional income by the companies in countries where currently a majority of Orphan Drugs are simply not marketed at all. Ideally. expanded penetration of the market and extra sales should allow to increase R&D funding as well as to decrease the overall level of spending in higher income countries. Negotiations centralized on the EU level may also help to develop well controlled and consistent technical mechanisms allowing to differentiate the effective price paid for Orphan Drugs in “lower-income EU clusters”. Such mechanisms could be based on the simple rebates/pay-back mechanisms. but more ambitiously. may as well combine some elements of the already run by EU in cooperation with pharma industry programs directed to support the R&D pharma development. 

Certainly. the ideas drafted above sound ambitious and would require a lot of understanding and trust from all stakeholders. but may offer many advantages in the currently fast-changing environment and therefore may be worth considering. Expanding such mechanisms globally or at least to the US may be even more difficult; still. as the US and EU form the majority of the global Orphan Drug market and are the main source of R&D investment in this segment. such a scenario is worth of considering as well.

### Limitations

Similar to other cross-country price-comparison studies. our study has many limitations. Our price comparisons are made on a per-brand basis and are not based on the real-world usage data. Therefore. its relation to the level of the total drug expenses should be interpreted with caution. Additionally. the selection of the lowest per mg/i.u. of the active substance price from all available presentations may not reflect real life practice. but as we used the same methodology for all cross-countries comparison. we consider this solution as the best available. 

The selection of the list prices (EU) and of VAFFS prices (US) for comparison is another limitation already discussed in the earlier parts of this article. However. as the US VAFSS prices used in our analysis are commonly considered as the lowest prices in the US and as the EU List Prices are per definition the highest paid in EU countries. the significant premium of US prices versus EU indicated in our study could be significantly higher in the real world. thus making our conclusions even stronger.

Findings of our study apply directly to the VA market. which has 9 million enrolled participants and 2018 pharmaceutical budget of approximately 11 billion USD [37]. This is relatively small compared to total of 360 billion USD spent on prescription drugs in the US. with Medicare accounting for 59.9 million participants and 94.7 billion USD in drug reimbursement costs in Part D [38] or Medicaid with 33.4 billion USD in drugs costs [16]. As in the recent studies. VA prices were indicated to be approximately 44% lower than prices paid by Medicare Part D30 or 48% lower than the published AWP [28]. which were commonly used as the initial point of reference in other studies. our findings confirm that price differences observed in other drug segments are also present in the Orphan Drugs segment. 

Due to the limitation of resources. we did not analyze the price differences between drugs with Orphan Drug status in US and without such status in EU. nor did we analyze the very significant difference in the number of the Orphan Drug designations and approvals between US and EU. with 1264 designations and 133 approvals granted by the end of 2015 by EMA in comparison to 3082 designations and 415 approvals by FDA [39]. 

## 5. Conclusions

US Orphan Drugs prices were higher than in the compared EU countries. 

Price differentials were smaller for the drugs which due to its characteristics (single and identical form marketed globally) are easier to compare across the countries or/and were introduced into the market more recently. This observation seems valid for both comparisons performed in our study—US vs. EU and EU cross-country. While the prices across EU countries seemed highly homogeneous. the number of the reimbursed and therefore available to patients Orphan Drugs in the EU country varied and was strongly and positively correlated with the economic power (GDP per capita) of the country. 

The above findings suggest that the eventual introduction of the price reference mechanisms currently reviewed by the US House of Representatives may generate significant savings in US drugs expenditure but may also put significant upward pressure on Orphan Drugs prices in EU. The already suggested public initiatives of the centralization of the pricing negotiations for innovative expensive treatments on the level of the entire EU may not only prevent such potential development but may also offer a win-win opportunity for all involved parties reassuring attractive return on R&D investment to the industry through widening patients’ access to the highly needed innovative treatments. thanks to well-structured mechanisms diminishing overall financial impact on less economically developed countries. 

Having said that. it is worth noting that the segment of orphan drugs is distinguishable from other segments of the pharmaceutical industry in that its product has some characteristics of a public good. This stems from the fact that variable costs are virtually insignificant in relation to the sunk cost of launching the product onto the market. In a broader economic perspective. optimal mechanism for fundings (almost) non-rivalrous goods are far from uniform price or uniform contribution per unit of consumption. This in turn entails a need for seeking alternative ways of financing the development and marketing of orphan drugs. and coordination of national drug policies. This is certainly a very challenging project for numerous reasons that are beyond the scope of this paper; however. we believe that the results of our study might facilitate such initiatives.

Independent of the above conclusions. our findings suggest that there may be a significant space for savings through more aggressive price negotiations in all analyzed countries in the field of Orphan Drugs with longer presence on the market. Generated funding could be used to accelerate the introduction of the new treatments for patients with rare diseases and provide additional incentive for R&D investment.

## Figures and Tables

**Table 1:** Orphan Drugs Analysis—used segmentation. US vs. 6 EU Countries.

Orphan Drugs with First FDA Approval in Years 1996–2014			Orphan Drugs with First FDA Approval in Years 2015–2019	
Single and Identical Pack Only	Brand Name	International non-proprietary name (INN)	Brand Name	International non-proprietary name (INN)
	Adcetris	brentuximab vedotin	Brineura	cerliponase alfa
	Gazyvaro (Gazyva in US)	obinutuzumab	Defitelio	defibrotide
	Hetlioz	tasimelteon	Epidyolex	cannabidiol
	Increlex	mecasermin	Galafold	migalastat
	Mozobil	plerixafor	Kanuma	sebelipase alfa
	Mylotarg	gemtuzumab ozogamicin	Kymriah	tisagenlecleucel
	Naglazyme	galsulfase	Lutathera	lutetium (177Lu) oxodotreotide
	Soliris	eculizumab	Luxturna	voretigene neparvovec
	Tobi Podhaler	tobramycin (podhaler)	Onpattro	patisiran
	Vimizim	elosulfase alfa	Oxervate	cenegermin
	Xagrid (Agrylin in US)	anagrelide	Spinraza	nusinersen
			Tegsedi	inotersen
			Xospata	gilteritinib
			Yescarta	axicabtagene ciloleucel
Other As Single and Identical Pack Only	Adempas	riociguat	Besponsa	inotuzumab ozogamicin
	Aldurazyme	laronidase	Cablivi	caplacizumab
	Alprolix	eftrenonacog alfa	Cresemba	isavuconazole
	Arranon (Atriance in EU)	nelarabine	Cresemba (inj)	isavuconazole inj
	Blincyto	blinatumomab	Crysvita	burosumab
	Carglumic acid (Carbaglu in EU)	carglumic acid	Darzalex	daratumumab
	Cerdelga	eliglustat	Diacomit	stiripentol
	Cometriq/Cabometyx	cabozantinib	Farydak	panobinostat
	Elaprase	idursulfase	Ruzurgi (Firdapse in EU)	amifampridine
	Esbriet	pirfenidone	Factor IX (Idelvion in EU)	albutrepenonacog alfa
	Fabrazyme	agalsidase beta	Natpar	parathyroid hormone
	Firazyr	icatibant	Ninlaro	ixazomib
	Iclusig	ponatinib	Ocaliva	obeticholic acid
	Imbruvica	ibrutinib	Onivyde pegylated liposomal	irinotecan hydrochloride trihydrate
	Pomalyst (Imnovid in EU)	pomalidomide	Kolbam (Orphacol in EU)	cholic acid
	Kalydeco	ivacaftor	Palynziq	pegvaliase
	Kuvan	sapropterin	Prevymis	letermovir
	Kyprolis	carfilzomib	Prevymis inj	letermovir inj
	Myalepta	metreleptin	Qarziba	dinutuximab beta
	Lumizyme (Myozyme in EU)	alglucosidase alfa	Rydapt	midostaurin
	Nexavar	sorafenib	Strensiq	asfotase alfa
	Ofev	nintedanib	Symdeko/Symkevi	ivacaftor/tezacaftor
	Opsumit	macitentan	Takhzyro	lanadelumab
	Pedea	ibuprofen	Tepadina	thiotepa
	Procysbi	mercaptamine, cysteamine	Vyndamax/Vyndaqel	tafamidis
	Gattex (Revestive in EU)	teduglutide	Wakix	pitolisant
	Revlimid	lenalidomide	Xermelo	telotristat ethyl
	Promacta (Revolade in EU)	eltrombopag	Yondelis	trabectedin
	Sirturo	bedaquiline	Zejula	niraparib
	Somavert	pegvisomant		
	Sylvant	siltuximab		
	Tasigna	nilotinib		
	Thalidomide	thalidomide		
	Trisenox	arsenic trioxide		
	Ventavis	iloprost		
	Vpriv	velaglucerase alfa		
	Vyxeos liposomal	daunorubicin/cytarabine		
	Galzin (Wilzin in EU)	zinc		
	Signifor/Signifor LAR	pasireotide		

**Table 2:** Price Data Sources.

Country	Data Source	Link	Data Sourced
Denmark	Laegemiddelstyrelsen, Danish Medines Agency	https://www.medicinpriser.dk/	17 February 2020
France	Ministère des Affairs sociales et de la Santé Agence Technique de l’information sur l’hospitalisation	http://medicprix.sante.gouv.fr/medicprix/ https://www.atih.sante.fr/unites-communes-de-dispensation-prises-en-charge-en-sus	18 March 2020
Germany	ABDA-Database, Pharmazie.com/IFA GmbH, Informationsstelle für Arzneispezialitäten GmbH	https://go.pharmazie.com/en/drug-databases/ http://www.ifaffm.de/de/ifa-gmbh/firmenportraet.html	18 August 2020
Greece	Greece Ministry of Health	https://www.moh.gov.gr/articles/times-farmakwn/deltia-timwn/6583-deltio-anathewrhmenwn-timwn-farmakwn-anthrwpinhs-xrhshs-dekembrioy-2019	25 February 2020
Poland	Poland Ministry of Health National Health Fund (NFZ)	https://www.gov.pl/web/zdrowie/refundacja3 https://www.nfz.gov.pl/aktualnosci/aktualnosci-centrali/	10 February 2020
Spain	Vidal Vademecum Spain	https://www.vademecum.es/productos-vademecum	23 February 2020

**Table 3:** Orphan Drugs price comparison: US vs. 6 EU Countries (Denmark, France, Germany, Greece, Spain, Poland).

Country	Compared Orphan Drugs *		Orphan Drugs “Single and Identical Pack Only” **		Orphan Drugs Other as Single and Identical Pack Only **		Total Number of Analyzed Pack Types
	Price Ratios (Quantity)	Price Ratio (Value)	Price Ratios (Quantity)	Price Ratio (Value)	Price Ratios (Quantity)	Price Ratio (Value)	
US ^1^	95		25		70		221
Germany ^2^	91	1.49	24	1.38	67	1.53	223
Denmark ^3^	83	1.46	20	1.11	63	1.57	164
Spain ^4^	60	1.57	15	1.44	45	1.61	96
France ^5^	54	1.86	15	1.54	39	1.97	110
Greece ^6^	49	1.81	10	1.81	39	1.81	92
Poland ^7^	26	2.11	6	1.69	20	2.24	52
Combined 6 EU Countries	363	1.64	90	1.43	273	1.71	958

* All drugs approved in US with Orphan Drug status by FDA and approved in EU with Orphan Drug status by EMA; with prices set in compared countries. ** Orphan Drugs with only one and identical pack type (same presentation, concentration, dose, number of tablets/capsules/vials in one pack) in US and all 6 EU countries. ^1^ US Department Veterans Affairs, https://www.va.gov/opal/nac/fss/pharmPrices.asp, accessed on 1 March 2020. ^2^ Pharmazie.com based on ABDA-Database, IFA (Informationsstelle für Arzneispezialitäten GmbH), https://go.pharmazie.com/en/drug-databases/, accessed on 1 July 2020. ^3^ Laegemiddelstyrelsen, Danish Medines Agency, https://www.medicinpriser.dk/, accessed on 1 March 2020. ^4^ Vidal Vademecum Spain, https://www.vademecum.es/productos-vademecu, accessed on 1 March 2020. ^5^ Ministère des Affairs sociales et de la Santé Agence Technique de l’information sur l’hospitalisation, http://medicprix.sante.gouv.fr/medicprix/, accessed on 1 March 2020, https://www.atih.sante.fr/unites-communes-de-dispensation-prises-en-charge-en-sus, accessed on 1 March 2020. ^6^ Greece Ministry of Health https://www.moh.gov.gr/articles/times-farmakwn/deltia-timwn/6583-deltio-anathewrhmenwn-timwn-farmakwn-anthrwpinhs-xrhshs-dekembrioy-2019, accessed on 1 December 2019. ^7^ Poland Ministry of Health, National Health Fund (NFZ), https://www.gov.pl/web/zdrowie/refundacja3, accessed on 1 December 2019, https://www.nfz.gov.pl/aktualnosci/aktualnosci-centrali/, accessed on 1 December 2019.

**Table 4:** Price Ratios EU Individual Country/Average Price from six EU Countries. Distribution.

	All Orphan Drugs (n = 93)			Single & Identical Pack Only (n = 25)			Other as Single & Identical Pack Only (n = 68)		
Price Ratio Range	2001–2014	2015–2019	2001–2019	2001–2014	2015–2019	2001–2019	2001–2014	2015–2019	2001–2019
<0.7	8	3	11	1	0	1	7	3	10
07–0.8	13	6	19	2	0	2	11	6	17
0.8–0.9	41	15	56	9	5	14	32	10	42
0.9–1.1	106	101	207	28	34	62	78	67	145
1.1–1.2	25	9	34	4	1	5	21	8	29
1.2–1.3	13	8	21	2	2	4	11	6	17
>1.3	13	2	15	2	0	2	11	2	13
All Price Ratios	219	144	363	48	42	90	171	102	273
<0.7	4%	2%	3%	2%	0%	1%	4%	3%	4%
07–0.8	6%	4%	5%	4%	0%	2%	6%	6%	6%
0.8–0.9	19%	10%	15%	19%	12%	16%	19%	10%	15%
0.9–1.1	48%	70%	57%	58%	81%	69%	46%	66%	53%
1.1–1.2	11%	6%	9%	8%	2%	6%	12%	8%	11%
1.2–1.3	6%	6%	6%	4%	5%	4%	6%	6%	6%
>1.3	6%	1%	4%	4%	0%	2%	6%	2%	5%

## Data Availability

Not applicable.
